# A Meta-Analysis of Randomised Placebo-Controlled Treatment Trials for Depression and Anxiety in Parkinson’s Disease

**DOI:** 10.1371/journal.pone.0079510

**Published:** 2013-11-13

**Authors:** Lakkhina Troeung, Sarah J. Egan, Natalie Gasson

**Affiliations:** School of Psychology and Speech Pathology, Curtin University, Perth, Australia; Baylor College of Medicine, United States of America

## Abstract

**Background:**

Psychopharmacotherapy currently constitutes the first-line treatment for depression and anxiety in Parkinson’s disease (PD) however the efficacy of antidepressant treatments in PD is unclear. Several alternative treatments have been suggested as potentially more viable alternatives including dopamine agonists, repetitive transcranial magnetic stimulation, and cognitive behavioural therapy (CBT).

**Method:**

A meta-analysis of randomised placebo-controlled trials for depression and/or anxiety in PD was conducted to systematically examine the efficacy of current treatments for depression and anxiety in PD.

**Results:**

Nine trials were included. There was only sufficient data to calculate a pooled effect for antidepressant therapies. The pooled effect of antidepressants for depression in PD was moderate but non-significant (*d* = .71, 95% CI = −1.33 to 3.08). The secondary effect of antidepressants on anxiety in PD was large but also non-significant (*d* = 1.13, 95% CI = −.67 to 2.94). Two single-trials of non-pharmacological treatments for depression in PD resulted in significant large effects; Omega-3 supplementation (*d* = .92, 95% CI = .15 to 1.69) and CBT (*d* = 1.57, 95% CI = 1.06 to 2.07), and warrant further exploration.

**Conclusions:**

There remains a lack of controlled trials for both pharmacological and non-pharmacological treatments for depression and anxiety in PD which limits the conclusions which can be drawn. While the pooled effects of antidepressant therapies in PD were non-significant, the moderate to large magnitude of each pooled effect is promising. Non-pharmacological approaches show potential for depression in PD however more research is required.

## Introduction

Parkinson’s disease (PD) is classically defined as a motor disorder of neurological aetiology. However, there has been a recent movement towards a reconceptualisation of PD in recognition of the multitude of cognitive and psychiatric disturbances that also feature in the disease [1]. Depression and anxiety are the two most clinically significant psychological disturbances in PD and affect up to half of all individuals with PD at some stage of their illness [2]. Research has shown that both depression and anxiety are among the greatest predictors of poor quality of life in PD and consistently rated as more detrimental to well-being and functional ability than motor symptoms [3], even in the most advanced stages of disease where motor symptoms have fully progressed [4].

Despite this, the majority of cases of depressive and anxiety disorders in PD are not effectively managed, and only an estimated 20% of people with PD experiencing depressive and/or anxiety complications receive some form of professional treatment [5]. A knowledge deficit has been identified as an underlying factor, with researchers and clinicians alike calling out for more evidence-based information to guide clinical efforts.

Currently, psychopharmacotherapy constitutes the first-line treatment for depression and anxiety in PD, with the two most traditionally administered psychiatric medications in PD being selective serotonin reuptake inhibitors (SSRIs) and tricyclic antidepressants (TCAs) [6]. There have been several uncontrolled trials [7]–[13] and randomised controlled trials (RCT) [14]–[23] of antidepressants in PD to date, however, the efficacy of antidepressant treatments in PD remains unclear. In the most recent meta-analysis of antidepressant treatments for depression in PD, Rocha et al. [24] reported a slightly better response rate associated with SSRIs relative to placebos (risk ratio  = 1.20) across five placebo-controlled RCTs, however this result was not statistically significant (95% CI = .57 to 2.52). A superior response rate was found for TCAs relative to SSRIs for depression in PD (RR = 1.78, 95% CI = 1.06 to 2.99) although this comparison was only based on two trials. There have been no trials specifically examining the effect of SSRIs or TCAs on anxiety as a primary outcome in PD. In addition, antidepressant treatment regimens in PD are complicated by concerns regarding polypharmacy [25], [26] and safety [27].

Consequently, there has been an emerging interest in the utility of alternative and non-pharmacological treatments for depression and anxiety in PD in recent years. Several alternate treatments have been suggested as safer and potentially more effective alternatives than antidepressant therapies and include dopamine agonists [28]–[34], Omega-3 fatty-acid supplementation [35]–[37], repetitive transcranial magnetic stimulation (rTMS) [38]–[43], and cognitive behavioural therapy (CBT) [44]–[55].

The aim of this meta-analysis is to provide a systematic evaluation of the efficacy of existing treatments for depression and/or anxiety in PD to better inform clinical care and future research. There have been four published meta-analyses for depression in PD to date [24], [56]–[58], while there have been no meta-analyses for anxiety in PD. A fifth meta-analysis of antidepressant therapies in PD was identified [59], however this study did not specifically examine the treatment of clinical depression in PD (i.e., a DSM or ICD diagnosis of depression was not a required inclusion criteria). Of the four existing meta-analyses specifically for depressive disorders in PD, all have focused solely on the efficacy of antidepressant interventions. The present study is therefore the first meta-analysis of both pharmacological and non-pharmacological treatments for depression in PD, as well as the first meta-analysis of any kind for anxiety in PD. To facilitate comparison of the treatment effect across different treatment modalities, the present analysis will include only non-active or placebo-controlled RCTs (no treatment, waitlist control, treatment as usual, clinical monitoring, pill).

## Methods

### Search Strategy

A comprehensive literature search was conducted for treatment studies of depression and/or anxiety in PD. Several online databases including Medline, PubMed, PsycInfo, Proquest, Cochrane Library, and EMBASE were systematically searched from the first available year of publication to July 2013 for the keywords; *Parkinson’s disease, depression, anxiety, treatment, therapy, RCT, trial.* Reference lists of previous meta-analyses of treatments for depression in PD were also searched.

### Study Selection

To be eligible for inclusion in the meta-analysis, studies had to:

1. Feature participants with idiopathic PD
2. With a DSM or ICD diagnosis of depression and/or anxiety
3. Evaluated an intervention targeting depression and/or anxiety in PD as the primary focus
4. Used a randomised controlled design with a non-active control (no treatment, treatment as usual, clinical monitoring, waitlist, placebo)
5. Employed a standardised primary outcome measure for depression and/or anxiety
6. Included sufficient quantitative data from which an effect size could be computed
7. Be written in English

### Data Extraction

Data from each study was extracted independently by two authors. The information extracted from each study were participants, interventions, comparisons, outcomes, and study design (PICOS), and pretreatment and posttreatment means and standard deviations for any depression and/or anxiety outcome measure where reported. Where all required means and standard deviations were not reported, *t*-values, *F* statistics or probability values reported for between-group comparisons on posttreatment scores were extracted to calculate effect sizes. Disagreements between authors on the eligibility of studies were discussed with a third author to determine a consensus.

### Risk of Bias Assessment

Eligible studies were assessed for risk of bias using the Cochrane Collaboration’s tool for assessing risk of bias [60]. The Cochrane tool classes studies as having low, moderate, or high risk of bias across six domains; sequence generation, allocation concealment, blinding, missing data, selective reporting and other biases.

### Statistical Analysis

All aspects of this meta-analysis were conducted in line with recommendations by the Preferred Reporting Items for Systematic Reviews and Meta-Analyses (PRISMA) statement [61]. Statistical analyses were guided by recommendations by Borenstein, Hedges, Higgins and Rothstein [62], DerSimonian and Kacker [63] and Marin-Martinez and Sanchez-Meca [64].

**Effect size calculation.** Effect sizes for each study were represented by the standardised mean difference (Cohen’s *d*) between the treatment and control groups at posttreatment. Where there was sufficient data reported (pretreatment and posttreatment means and standard deviations for treatment and control groups), effect sizes were calculated using change scores. Where all the required data were not reported, effect sizes were computed using *t*-values, *F* statistics or probability values reported for between-group comparisons on posttreatment scores. Hedge’s small sample size correction [65] was applied to effect size estimates where the number of participants was less than 20. Finally, where a study included two treatment conditions against a single control, participants in the control condition were split evenly into two subgroups to serve as controls for each treatment condition.

**Pooled effect size calculation.** A random-effects model was used to calculate pooled effect sizes. Individual effect sizes from each study were weighted to account for both within-study and between-study variance using the Hedges and Vevea [66] ‘weighting by inverse variance’ method. Between-study variance was calculated using the Dersimonian and Laird [67] general method of moments estimate. The pooled effect size was calculated by dividing the sum of weighted effect sizes by the sum of the individual weights of each study. To test the statistical significance of individual and pooled effect sizes, 95% confidence intervals were calculated as per Borenstein et al. [62].

**Publication bias.** Publication bias refers to the tendency for researchers to publish only positive results from clinical trials while neglecting to report any small or non-significant findings which can ultimately result in inflated pooled effect size in meta-analyses [68]. Publication bias in the current meta-analysis was assessed using Egger’s [69] regression asymmetry test and Rosenthal’s [70] Fail-Safe *N* method.

**Heterogeneity analysis.** Heterogeneity within a meta-analysis is undesirable as it indicates that a treatment has differential effects across studies [71]. Heterogeneity in the current meta-analysis was assessed using the *I* ^2^ statistic [72], which is an index of the proportion of total variance in the pooled effect size that is due to heterogeneity between studies.

## Results

### Search Results

A total of 86 treatment trials for depression and/or anxiety in PD were identified. Fifty-six papers were excluded as they were case studies or case series (n = 26), uncontrolled trials (n = 28), or non-randomised controlled trials (n = 3). This resulted in a total of 29 randomised controlled trials (see Table 1). Publication dates ranged from 1975 to 2012, with 72% of studies published over the past 10 years. There were 28 RCTs for the treatment of depression in PD, while one RCT was targeted at the treatment of comorbid depression and anxiety [73]. There were 16 RCTs of antidepressant interventions, 4 RCTs of antiparkinsonian medications, 5 RCTs for rTMS, 2 RCTs for CBT, and one RCT each of atomoxetine (selective norepinephrine reuptake inhibitor) and Omega-3 supplementation.

**Table 1:** List of Randomised Controlled Trials for Depression and/or Anxiety in PD.

Intervention Type	First Author (Year)	Treatment	Comparison	*N*
Antidepressant	Alca (2011)	Sertraline (SSRI)	Venlafaxine (SNRI)	32
	Andersen (1980)	Nortriptyline (TCA)	Placebo	22
	Antonini (2006)	Sertraline (SSRI)	Amitriptyline (TCA)	31
	Avila (2003)	Nefazodone (SARI)	Fluoxetine (SSRI)	16
	Dell’Agnello (2001)	Fluoxetine (SSRI)	Fluvoxamine (SSRI)	62
			Citalopram (SSRI)	
			Sertraline (SSRI)	
	Devos (2008)	Citalopram (SSRI)	Placebo	48
		Desipramine (TCA)		
	Djokic (2010)	Clomipramine (TCA)	Placebo	339
		Fluoxetine (SSRI)		
		Sertraline (SSRI)		
		Escitalopram (SSRI)		
		Mirtazapine (NaSSa)		
		Tianeptine (SSRE)		
	Leentjens (2003)	Sertraline (SSRI)	Placebo	12
	Menza (2009)	Nortriptyline (TCA)	Placebo	52
		Paroxetine (SSRI)		
	Rabey (1996)	Fluvoxamine (SSRI)	Amitriptyline (TCA)	47
	Richard (2012)	Paroxetine (SSRI)	Placebo	115
		Venlafaxine (SNRI)		
	Serrano-Duenas (2002)	Fluoxetine (SSRI)	Amitriptyline (TCA)	77
	Trivedi (2003)	Sertraline (SSRI)	Bupropion	46
	Wermuth (1998)	Citalopram (SSRI)	Placebo	18
	Werneck (2009)	Trazodone (SARI)	Placebo	20
	Xia (2012)	Fluoxetine (SSRI)	Fluoxetine (SSRI) + Eelectroacupuncture	60
				
Pharmacological	Barone (2006)	Pramipexole (DA)	Sertraline (SSRI)	67
(other)	Barone (2010)	Pramipexole (DA)	Placebo	287
	Rektorova (2003)	Pramipexole (DA)	Pergolide (DA)	41
	Steur (1997)	Moclobemide (MAO-I)	Moclobemide + SSelegiline	10
	Weintraub (2010)	Atomoxetine (SNRI)	Placebo	55
				
Supplements	Moralez Da Silva (2008)	Omega-3 fatty-acid	Placebo	29
				
rTMS	Boggio (2005)	Left prefrontal rTMS	Fluoxetine (SSRI)	25
	Cardoso (2008)	Left prefrontal rTMS	Fluoxetine (SSRI)	21
	Fregni (2004)	Left prefrontal rTMS	Fluoxetine (SSRI)	42
	Fregni (2006)	Left prefrontal rTMS	Fluoxetine (SSRI)	26
	Pal (2010)	Left prefrontal rTMS	Placebo (sham rTMS)	22
				
Psychotherapy	Dobkin (2011)	Individual CBT	Clinical monitoring	80
	Veazey (2009)	Telephone CBT	Telephone counselling	10
				

TCA  =  tricyclic antidepressant, SSRI  =  selective serotonin reuptake inhibitor, SARI  =  serotonin 2 antagonist/reuptake inhibitor, DA  =  dopamine agonist, SNRI  =  selective norepinephrine reuptake inhibitor, rTMS  =  repetitive transcranial magnetic stimualation, CBT  =  cognitive behavioural therapy.

**Figure 1:**
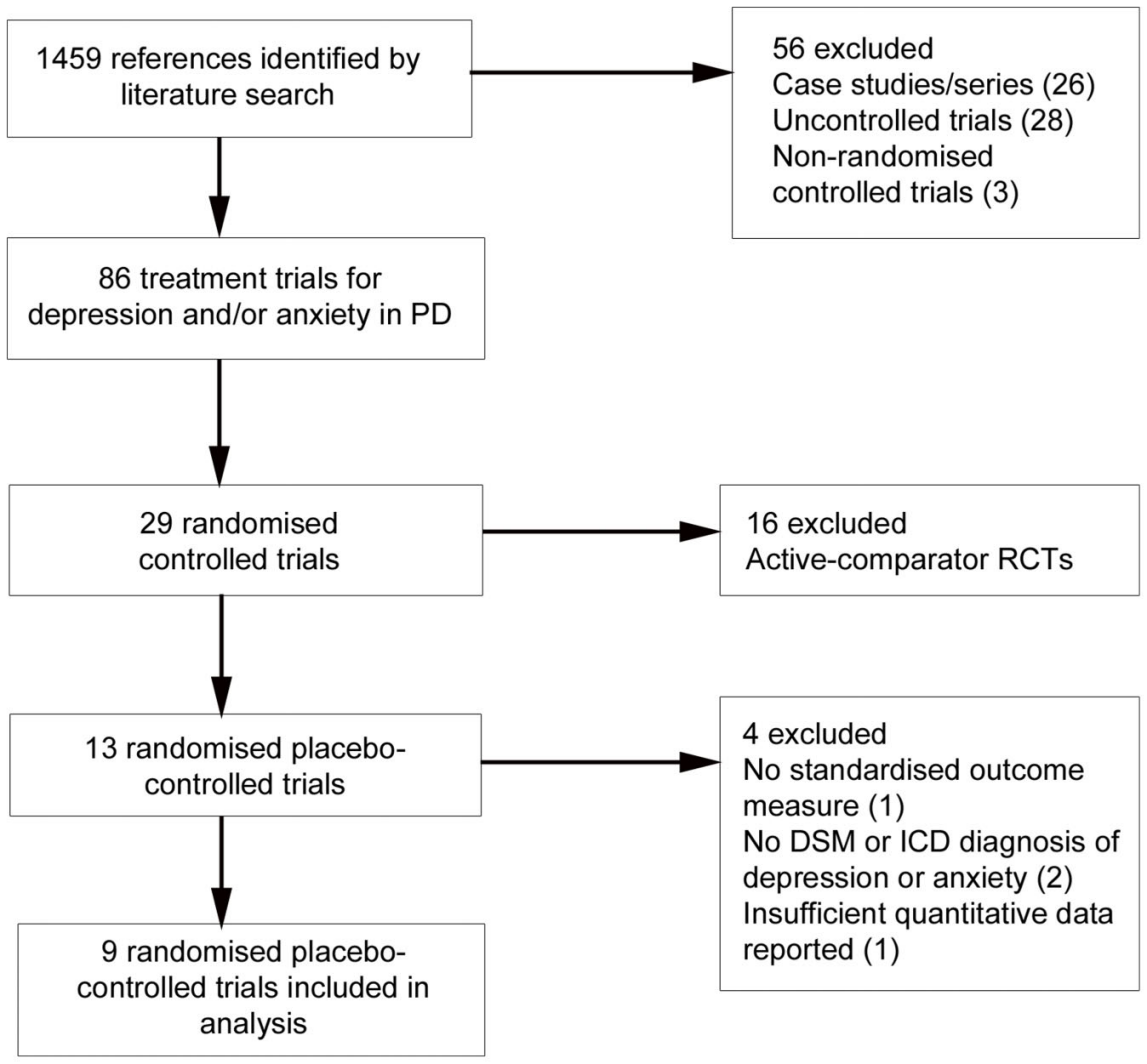
Flowchart of inclusion of studies.

Of the 29 RCTs, 16 were excluded as these were active-comparator trials, leaving 13 placebo-controlled RCTs. A further four placebo-controlled trials were excluded. The Andersen et al. [15] trial of the TCA nortriptyline was omitted for not using a standardised outcome measure. The Barone et al. [33] trial of the dopamine agonist pramipexole and Werneck et al. [74] trial of trazodone (serotonin antagonist and reuptake inhibitor) were excluded as participants in both studies were not required to have a clinical diagnosis of depression according to DSM or ICD criteria. Finally, The Djokic et al. [75] trial comparing six antidepressants with placebo was also excluded as there was insufficient quantitative data in the published conference abstract to calculate effect sizes. Figure 1 presents a flowchart describing the inclusion process.

Two active-comparator RCTs have been previously included in an earlier meta-analysis [56] of placebo-controlled RCTs of SSRIs in PD. The authors argued that the low dosage of the comparator amitriptyline drug in the Antonini et al. [16] study, as well as the unestablished nature of the rTMS treatment in the Fregni et al. [42] trial were equivalent to placebo-controlled conditions. These two trials were not included in the main analysis for this study as they were not explicit placebo-controlled trials, however they were included as part of the subsequent sensitivity analysis to examine whether their inclusion would significantly alter the results of the main analyses.

### Study Characteristics

The 9 eligible studies included 450 participants (252 in treatment conditions, and 180 in control conditions). There were five RCTs of antidepressants for depression in PD [17]–[20], [22], and one RCT each of Omega-3 fatty-acid supplementation [37], atomoxetine [76], rTMS [43], and CBT [55]. There were no eligible RCTs of dopamine agonists or ECT for depression in PD, and no new placebo-controlled RCTs of antidepressants for depression in PD published since the Rocha et al. [24] meta-analysis. Table 2 summarises the characteristics of the trials included in the present analysis.

**Table 2:** Characteristics of Placebo-Controlled Randomised Controlled Trials for Depression in PD used in the Present Meta-Analysis.

First author	Year	Location	Condition	Design	Time (weeks)	Diagnostic system	*N*	Gender (% male)	Age (M, SD)	Duration of PD (M, SD)	Mean H&Y Stage	Primary Outcome
Devos	2008	France	Citalopram, Desipramine, Placebo	Double-blind parallel	4	DSM-IV	48	21 (43.5)	64.7 (6.5)	8.1	II	MADRS
Dobkin	2011	USA	CBT, Clinical monitoring	Parallel	10	DSM-IV	80	48 (60)	64.6 (10.5)	6.3 (5.5)	II	HAM-D
Leentjens	2003	Netherlands	Sertraline, Placebo	Double blind parallel	10	DSM-IV	12	8 (66.6)	67 (7.8)	N/A	II	MADRS
Menza	2009	USA	Paroxetine, Nortriptyline, Placebo	Double-blind parallel	8	DSM-IV	52	27 (51.9)	62.2 (8.7)	6.1	II	HAM-D
Moralez Da Silva	2008	Brazil	Omega-3, Placebo	Double-blind parallel	12	DSM-IV	29	12 (42)	64.4	N/A	II	MADRS
Pal	2010	UK	rTMS, Placebo	Double-blind parallel	10 days	DSM-IV	22	11 (50)	68.5 (7.9)	6.25	II	MADRS
Richard	2012	USA	Sertraline, Venlafaxine, Placebo	Double-blind parallel	12	DSM-IV	115	73 (63.5)	63.5 (10.7)	4.9	II	HAM-D
Weintraub	2010	USA	Atomoxetine, Placebo	Double-blind parallel	8	DSM-IV	55	36 (66)	64.3 (10.5)	6.9 (6.2)	N/A	IDS-C
Wermuth	1998	Denmark	Citalopram, Placebo	Double-blind parallel	8	DSM-III-R	37	16 (43.2)	65.9 (17.5)	N/A	II	HAM-D

DSM-IV  =  Diagnostic and Statistical Manual of Mental Disorders (fourth edition), DSM-III-R  =  Diagnostic and Statistical Manual of Mental Disorders (third edition, revised), MADRS  =  Montgomery-Asberg Depression Scale, HAM-D  =  Hamilton Depression Inventory, IDS-C  =  Inventory of Depressive Symptomatology-Clinician.

### Risk of Bias Assessment

Six studies had low risk of bias (17, 19, 20, 37, 55, 76) and three had unclear risk of bias [18], [22], [43]. Of the three studies with unclear risk, two did not adequately describe sequence generation procedures [22], [43] and all three did not adequately describe allocation procedures. All studies were double-blinded (with the exception of the CBT trial [55] where blinding is not possible), and had low risk of attrition and reporting bias.

### Depression

**Figure 2:**
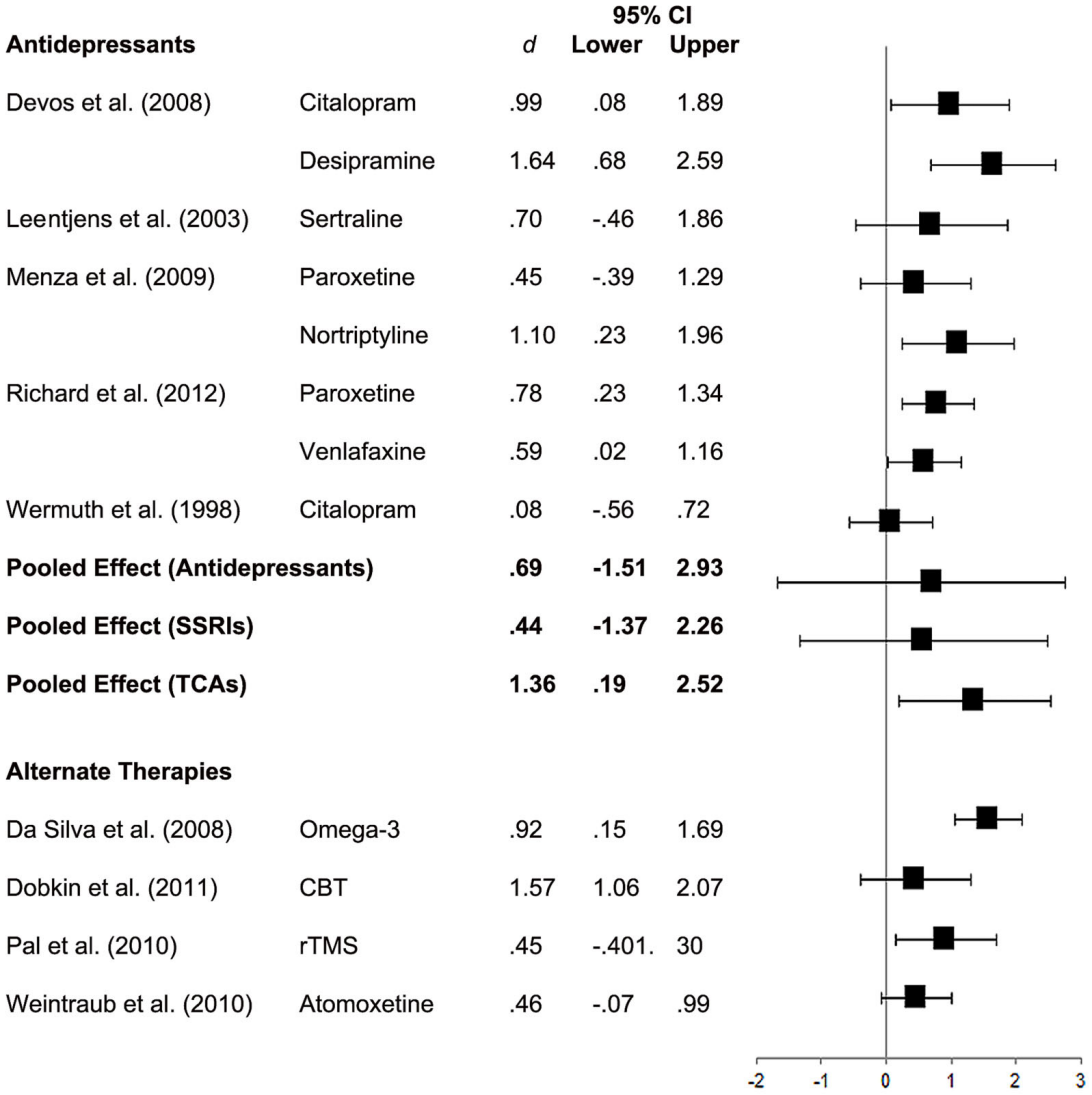
Forest Plot of Effect Sizes for Depression.

A forest plot of effect sizes and 95% confidence limits for all interventions appears in Figure 2. All interventions favoured the treatment condition at posttreatment with effect sizes ranging from.08 to 1.64. However, only 58% of the treatment effects were statistically significant. These were; citalopram and desipramine [17], nortriptyline [19], paroxetine and venlafaxine [20], Omega-3 supplementation [43], and CBT [55].

A pooled effect size was computed only for antidepressant interventions as this was the only treatment modality with more than one published placebo-controlled RCT. The pooled effect for antidepressants (*n* = 8) was moderate (*d* = .71) in favour of antidepressants, although this was not statistically significant (95% CI = –1.33 to 3.08). There was also substantial heterogeneity observed between the antidepressant studies, *I* ^2^ = 68.53%, *p* <.05. Egger’s regression test showed no evidence of publication bias among the antidepressant studies, *p* = .652. A fail-safe *N* statistic was not computed given that the pooled effect of antidepressants was non-significant.

**Sensitivity and Subgroup Analyses.** Given the significant heterogeneity observed across antidepressant studies, subgroup analyses were conducted for SSRIs and TCAs separately. The pooled effect for SSRIs was moderate and non-significant (*d* = .57, 95% CI = –1.33 to 2.47). The pooled effect for TCAs was significant and large (*d* = 1.35, 95% CI = .19 to 2.52). There was no longer any significant heterogeneity across studies for both SSRIs (*I* ^2^ = 0.00%, *p* >.05) and TCAs (*I* ^2^ = 21.13%, *p* >.05), suggesting that the initial heterogeneity was attributable to the different drug classes and mechanisms of action.

A further sensitivity analysis was conducted including data from two additional active-comparator antidepressant trials [16], [42] that were included in the Skapinakis et al. [56] meta-analysis of placebo-controlled RCTs of SSRIs for depression in PD. Inclusion of these two trials did not significantly alter findings. The pooled effect for antidepressants remained moderate and non-significant (*d* = .62, 95% CI = –2.13 to 3.37), as did the pooled effect for SSRIs (*d* = .48, 95% CI = –1.88 to 2.84), although there was a slight reduction in the magnitude of both pooled effects.

### Anxiety

There were no RCTs of any treatment for anxiety in PD, however, four of the depression trials reported the effect of treatment on anxiety as a secondary outcome. There were two trials of antidepressants [17], [19], one trial of CBT [55], and one trial of atomoxetine [76].

**Figure 3:**
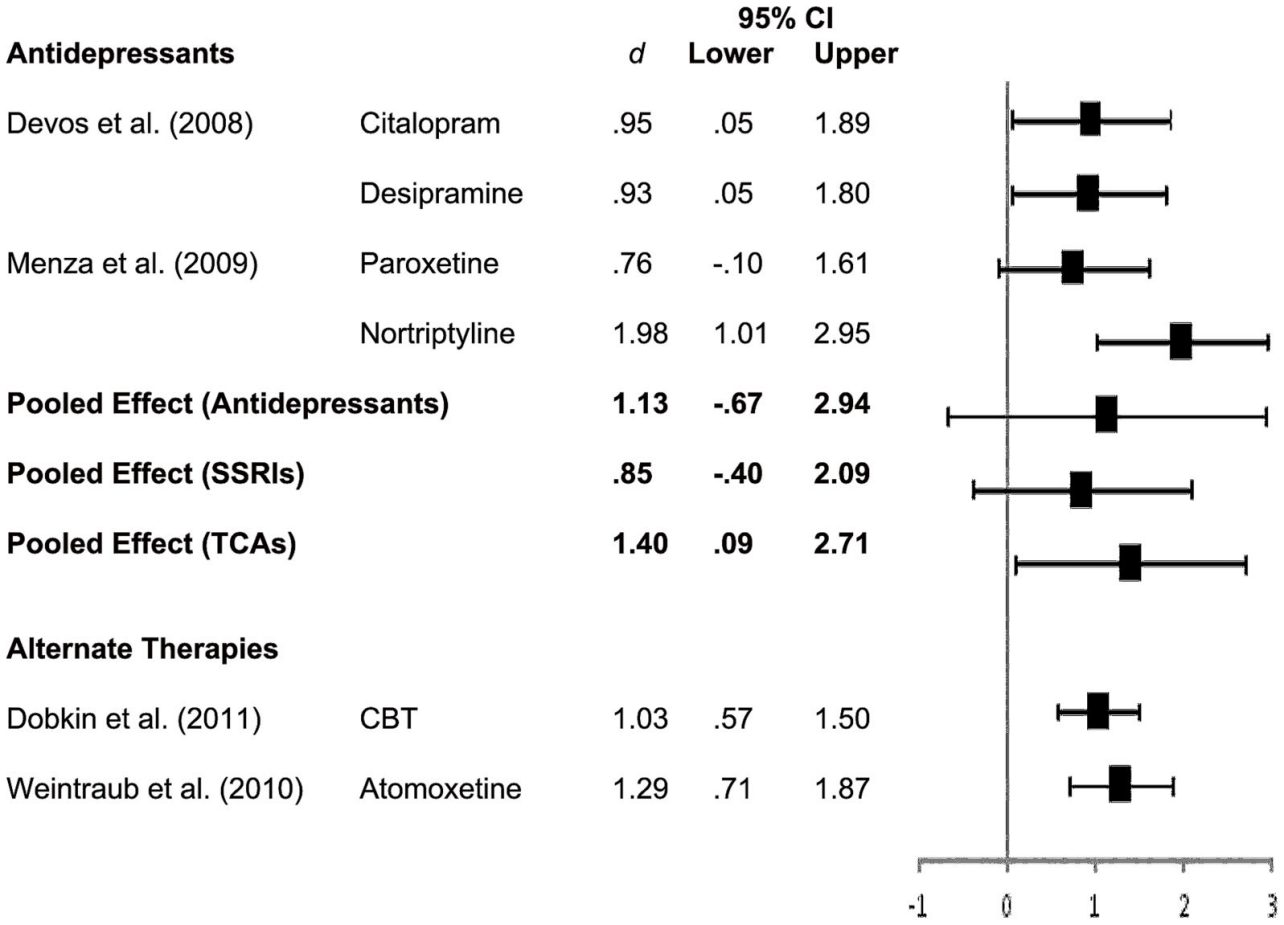
Forest Plot of Effect Sizes for Anxiety.

A forest plot of effect sizes and 95% confidence intervals for all interventions reporting the secondary effect of treatment on anxiety, as well as the pooled effect for antidepressants, SSRIs and TCAs appears in Figure 3. Apart from paroxetine which showed no significant effect on anxiety, all interventions resulted in large and statistically significant reductions in anxiety with effect sizes ranging from.93 to 1.98. A pooled effect size for antidepressants was computed by pooling data from the Devos et al. [17] and Menza et al. [19] trials. The pooled effect size for antidepressants on anxiety in PD was large (*d* = 1.13) but non-significant (95% CI = –.67 to 2.94). Again, there was substantial inconsistency in the effect of antidepressants on anxiety across studies, *I^2^* = 75.35%, *p* <.05. Subgroup analyses were conducted to examine the separate effect of SSRIs and TCAs on anxiety in PD. Similar to depression, the pooled effect for TCAs was large and significant (*d* = 1.40, 95% CI = .09 to 2.70) while the pooled effect for SSRIs was non-significant (*d* = .85, 95% CI = –.40 to 2.09). Overall, TCAs, atomoxetine and CBT all demonstrated a significant and large secondary effect on anxiety outcomes.

## Discussion

In the first published meta-analysis of treatments for depression in PD in 1995, Klaassen and colleagues [57] concluded that there was no available empirical evidence with which to base a treatment plan for depression in PD. While there have certainly been an increase in studies in years since this seminal review, it would appear that the empirical treatment literature for depression and anxiety in PD still requires further work. An extensive literature search revealed only nine trials meeting the inclusion criteria of being randomised placebo-controlled trials for depression and/or anxiety in PD, and employing formal diagnostic procedures and standardised outcome measures. Thus, despite the high prevalence of depression and anxiety in PD there is still a considerable lack of controlled research in the treatment of these disorders.

### Main Findings

This meta-analysis was the first to provide a controlled pooled effect size estimate for antidepressant therapies in PD. While there have been four prior meta-analyses assessing the efficacy of antidepressant therapies for depression in PD to date, none of these reviews calculated a pooled effect size of antidepressant therapies in PD. There was insufficient empirical data to do so at the time of the Klaassen et al. [57] review, while Weintraub and colleagues [58] pooled results from both RCTs and non-RCTs and reported an uncontrolled pooled effect size for antidepressant therapies in PD. In the two most recent meta-analyses, Skapinakis et al. [56] and Rocha et al. [24] both calculated and reported risk ratios for antidepressant response in PD rather than standardised treatment effects. The present analysis showed that the pooled effect of antidepressants for depression in PD was moderate but non-significant (*d* = .71, 95% CI = –1.33 to 3.08), as was the pooled effect for the current first-line SSRI treatments (*d* = .57, 95% CI = –1.33 to 2.47). The secondary effect of antidepressants on anxiety in PD was large with large pooled effect sizes observed for both antidepressant therapies in general (*d* = 1.13, 95% CI = –.67 to 2.94) and SSRI treatments (*d* = .85, 95% CI = –.40 to 2.09), although again, both results were non-significant.

The finding that both antidepressants in general and SSRI therapies have a non-significant pooled effect on depression in PD relative to placebo is consistent with prior results. Weintraub et al. [58] reported a large and statistically significant effect of antidepressants on depression in PD (*d* = .95, 95% CI = .76 to 1.14), however this effect was less than that for placebo for depression in PD (*d* = 1.18, 95% CI = .55 to 1.81) suggesting that overall there appears to be no benefit of antidepressant therapies relatiave to placebo, consistent with the present findings. Skapinakis and colleagues [56] reported a slightly better response rate associated with SSRIs relative to placebos, however this result was not statistically significant (RR = 1.08, 95% CI = .75 to 1.55). Most recently, Rocha et al. [24] reported an improved response rate associated with SSRI treatments relative to placebo following the publication of the largest RCT of antidepressants in PD to date [20] however again this result was not significant (RR = 1.20, 95% CI = .57 to 2.52).

While it has been suggested that this result indicates that the widespread use of antidepressants in PD is largely unjustified, the magnitude of the pooled effect obtained in this study suggests that antidepressant therapies show promise in the treatment of the depression in PD. A pooled effect size of.71 represents a moderate to large effect and indicates that individuals with PD treated with antidepressants do experience a reduction in depressive symptoms compared with placebo, even if this effect is statistically non-significant. Similar to the conclusions of Rocha et al. [24] and Skapinakis et al. [56], at this stage, it is likely that the non-significance of the pooled effect for antidepressants in PD reflects a Type II error due to the very limited number of available placebo-controlled RCTs in the literature at present (*n* = 5). Ultimately, there is a need for more controlled research to resolve the ambiguity surrounding the efficacy of antidepressant therapies in PD.

In regards to the comparative efficacy of SSRIs and TCAs, the results of this meta-analysis support the general consensus that TCAs are more effective than SSRIs for the treatment of depression in PD. The pooled effect for TCAs was large and significant for both depression and anxiety while the pooled effects for SSRIs were moderate but non-significant. Rocha et al. [24] also reported a superior response rate for depression associated with TCAs than SSRIs (RR = 1.78, 95% CI = 1.06 to 2.99). Despite the efficacy of TCAs in PD, the high side-effect profile associated with this class of medications has seen a sharp decline in its use in PD. Thus, SSRIs remain the first-line and most widely utilised treatment for depression in PD at present. It is therefore imperative to resolve the ambiguity surrounding the efficacy of SSRIs for depression in PD to ensure that individuals with PD are being offered the most optimal first-line treatment.

However, although the current results suggest that antidepressant therapies do show promise in reducing symptoms of depression and anxiety in PD, there still remains concerns regarding polypharmacy, adverse drug interactions, and harmful side effects. These concerns regarding safety have ultimately resulted in an emerging interest in the utility of non-pharmacological treatment approaches for depression and anxiety in PD in recent times. The present analysis was the first to systematically examine the efficacy of non-pharmacological therapies for depression and anxiety in PD, and highlighted the potential of several non-pharmacological treatments as viable alternatives to antidepressant therapies for depression and anxiety in PD.

Although these findings must be interpreted with caution as they are based on the results of single RCTs, at this stage it would appear that there are several treatment options that warrant further research as alternatives to antidepressant therapies. The trial of Omega-3 supplementation [37] resulted in a significant large effect on depressive symptoms (*d* = .92, 95% CI = .15 to 1.69), while the trial of atomoxetine [76] resulted in a significant large effect on anxiety symptoms (*d* = 1.29, 95% CI = .71 to 1.87). In addition, the CBT trial [55] resulted in the largest effect on depression in PD (*d* = 1.57, 95% CI = 1.06 to 2.07) over all other interventions including TCAs, as well as a large secondary effect on anxiety symptoms (*d* = 1.03, 95% CI = .57 to 1.50). While these results are promising, neither Omega-3 supplementation nor atomoxetine are established treatments for depression and/or anxiety in primary psychiatric populations at present. CBT, however, has extensive evidence for efficacy in the treatment of depression and anxiety in general psychiatric populations and general older adult populations [77], [78]. In recognition of the potential of CBT for depression in PD, Black [79] recently urged researchers and clinicians alike to truly consider the utility of this ‘new (old)’ treatment modality as an alternate to current pharmacological regimens for depression in PD.

### Limitations and Direction for Future Research

There are several limitations to this meta-analysis. First, the restriction of studies to only those in English language excludes trials conducted and reported in non-English speaking countries. The second and primary limitation of this meta-analysis concerns the limited number of studies available for inclusion for analysis which has important implications for the interpretation of findings. As previously noted, while current results indicate that the pooled effect of antidepressant therapies for the treatment of depression in PD is non-significant, this result may likely represent a Type-II error given that moderate to large effect sizes were observed. There is a pressing need for more well-designed placebo-controlled trials of SSRIs in PD to provide a more accurate estimate of treatment effect, especially given that such treatments currently constitute the first-line approach for depression and anxiety in PD.

There is also a need for research and treatment trials specifically for anxiety in PD. While the empirical literature for depression treatments in PD is steadily increasing, the dearth of empirical research on the treatment of anxiety disorders in PD was highlighted in this study, with no RCTs of any treatment intervention for anxiety in PD identified. While anxiety and depression share an overlap of symptoms, there are core components of anxiety disorders that are distinct from depression and that require specific clinical attention.

Finally, researchers developing treatments for depression and anxiety in PD should also consider the utility of non-pharmacological treatment approaches, particularly, CBT as a potential alternative to current pharmacological treatments. While pharmacological treatments have been heavily favoured for the treatment of depression in PD, CBT offers a safer alternative. Preliminary data indicates that CBT is an efficacious treatment for individuals with PD however again there is a need for more studies in order to establish the magnitude of treatment effect.

## Conclusions

Despite increased scientific awareness of the significance and impact of depression and anxiety in PD over the past decade, there remains a lack of controlled trials for both pharmacological and non-pharmacological treatments for depression and anxiety in PD to guide clinical care. The lack of controlled trials also limits the conclusions which can be drawn from this analysis. Based on the available results, it would appear that both pharmacological and non-pharmacological interventions show potential in the treatment of depression and anxiety in PD. While the pooled effects of antidepressant therapies in PD were non-significant, the moderate to large magnitude of the pooled effect for both depression and anxiety is promising. More controlled trials are required to establish a more valid and reliable estimate of the treatment effect of antidepressants in PD. CBT appears to be a particularly promising non-pharmacological approach, and the results of this meta-analysis strongly suggest that future research needs to also be directed at the development and evaluation of CBT interventions in PD.

## Supporting Information

Checklist S1 PRISMA Checklist
(DOC)
